# Tibial tunnel malposition is a risk factor for osteoarthritis following ACL reconstruction at long term follow up: a retrospective study

**DOI:** 10.1051/sicotj/2026003

**Published:** 2026-05-19

**Authors:** Julien Erard, Maxime Rarchaert, Luca Andriollo, Cécile Batailler, Sébastien Lustig, Elvire Servien

**Affiliations:** 1 Univ Lyon, Claude Bernard Lyon 1 University, IFSTTAR, LBMC UMR_T9406 F-69622 Lyon France; 2 Orthopaedics surgery and Sports Medicine Department, FIFA medical center of excellence, Croix-Rousse Hospital, Lyon University Hospital Lyon France; 3 Ortopedia e Traumatologia, Fondazione Poliambulanza Istituto Ospedaliero Brescia Italy; 4 LIBM – EA 7424, Interuniversity Laboratory of Biology of Mobility Claude Bernard Lyon 1 University Lyon France

**Keywords:** Tunnel position, CT-scan, ACL reconstruction, Osteoarthritis, Anterior cruciate ligament

## Abstract

*Purpose*: This study aimed to compare long-term radiographic OA development after ACLR according to tunnel positioning assessed on 3D postoperative CT scans. *Methods*: Tunnel positioning was studied using 3D reconstructions, and tunnels were considered malpositioned if femoral tunnels were classified as type II or III (F), and tibial tunnels had an anteroposterior (AP) gap ≥ 5 mm and/or a mediolateral (ML) gap ≥ 2 mm compared to the native ACL location. Advanced radiographic OA was stages C and D according to the IKDC classification. *Results*: In this retrospective study, 76 consecutive patients who underwent ACLR were evaluated at a mean follow-up of 10.4 years. The mean age was 34.0 years. At the last follow-up, 18.4% of the patients had an advanced radiographic stage of OA. The distribution of malpositioned tunnels was as follows: 30.3% F, 17.1% AP, 19.7% ML, 11.8% AP+ML, and 5.3% AP+ML+F. There was a significant association between AP+ML (*p* = 0.022) and AP+ML+F (*p* < 0.001) tunnels and the IKDC C/D group. *Conclusion*: The malpositioning of tibial tunnels, specifically the combination of ML and AP displacement, is significantly associated with an advanced radiographic stage of OA at a follow-up of 10 years, regardless of femoral tunnel malposition. *Level of Evidence*: Level II.

## Introduction

Anterior cruciate ligament (ACL) injury is a common orthopaedic injury. Incidence ranges between 60.9 and 78 per 100 000 person-years in the literature [1, 2]. This injury has significant implications, including post-traumatic osteoarthritis (OA) [3]. An anatomical ACL reconstruction (ACLR) with optimal tunnel positioning is essential to restore the knee’s native kinematics [4]. After ACL injury, irrespective of whether the patients were treated operatively or nonoperatively, the relative risk of developing OA increased [5]. Despite the significant number of ACLRs carried out each year, technical errors remain frequent and play a contributing role in failures [6]. The transtibial femoral tunnel positioning technique is less accurate [7] and has been associated with higher rates of OA at longterm follow up compared with the antero-medial approach for femoral tunnel positioning [8]. Tunnel malposition is one of the leading causes of technical errors and is widely observed among the reasons for ACL revisions [9]. Historically and in daily practice, radiography is widely used to analyze tunnel positioning. However, some authors highlight the lack of precision of these techniques [10]. Recently, several protocols using cross-sectional imaging have been described, allowing precise identification of bony landmarks and ACL footprints [11].

Previous studies reported an association between sagittal tibial tunnel position and radiographic OA [12], whereas others found no correlation [13]. Regarding femoral tunnel placement, previous studies also shown conflicting results [14, 15]. None of the studies evaluating long-term OA after ACL reconstruction used 3D reconstructions from postoperative CT scan to classify tunnel positioning.

The aim of this study was to compare long-term radiological development of OA after ACLR according to tunnel positioning, identified on 3D reconstructions from postoperative CT scan. The hypothesis was that a higher rate of radiological OA was associated with non-anatomical tunnel positioning at long- term follow-up.

## Methods

### Patients

Consecutive patients aged between 15 and 60 years old from a single institution undergoing primary ACLR between February 2012 and December 2015 were included in this retrospective study. Patients underwent arthroscopic ACLR using a single-bundle technique with an outside-in drilling technique in all cases. First, a diagnostic arthroscopy was performed, followed by a systematic assessment of the cartilage and menisci. Meniscus repair was performed whenever possible. Meniscal resection was only performed for vertical flap and radial tears in zone 3, complex lesions with degenerative tissue, or failed repairs. All data were collected retrospectively from the medical records.

As part of the standard practice of the department during this time period, CT scans were obtained within 6 weeks postoperatively to evaluate tibial and femoral tunnel positioning. Each CT scan was performed by the same imaging service. The scanner was a CR-Brilliance CT (cut thickness, 0.67 mm; increment, 0.33 mm). The images were processed using Centricity^®^ software (GE Medical Systems, Chicago, IL; 2006).

The evaluation of ACL tear was conducted clinically using the Lachman test and pivot shift test, with confirmation provided by an MRI. Exclusion criteria were patients with preoperative radiographic OA (IKDC stage B, C, or D) [16].

One patient was excluded for preoperative advanced OA on radiography. A total of 76 ACLRs were included in the study group with a mean follow-up of 10.4 years (IQR 9.9–11.1). Mean age at surgery was 34.0 years (IQR 24.1–46.5) with an average BMI of 23.2 kg/m^2^ (IQR 21.1–25.8). Grafts consisted of either BPTB (70; 92.1%), 4-strand HT (5; 6.6%), or QT (1; 1.3%). Medial or lateral menisectomy was performed for 18 (23.7%) patients. The presence of a cartilage lesion was described for 6 (7.9%) patients. Patient characteristics are summarized in Table 1.

**Table 1:** Cohort characteristics.

	Median (range)	*N* (%)
Population		76 (100%)
Age (years)	34.0 (15–60)	
Age at surgery > 30		40 (52.6%)
Gender		
Male		40 (52.6%)
Female		36 (47.4%)
BMI	23.2 (17–37)	
BMI > 30		5 (6.6%)
Side		
Right		37 (48.7%)
Left		39 (51.3%)
Graft		
Patellar tendon		70 (92.1%)
Hamstrings		5 (6.6%)
Quadriceps tendon		1 (1.3%)
Injury-to-surgery time > 16 months		16 (21.1%)
Meniscectomy		18 (23.7%)
Pivot sport		35 (46.1%)
Cartilage lesion		6 (7.9%)

### Tibial tunnel position

**Figure 1:**
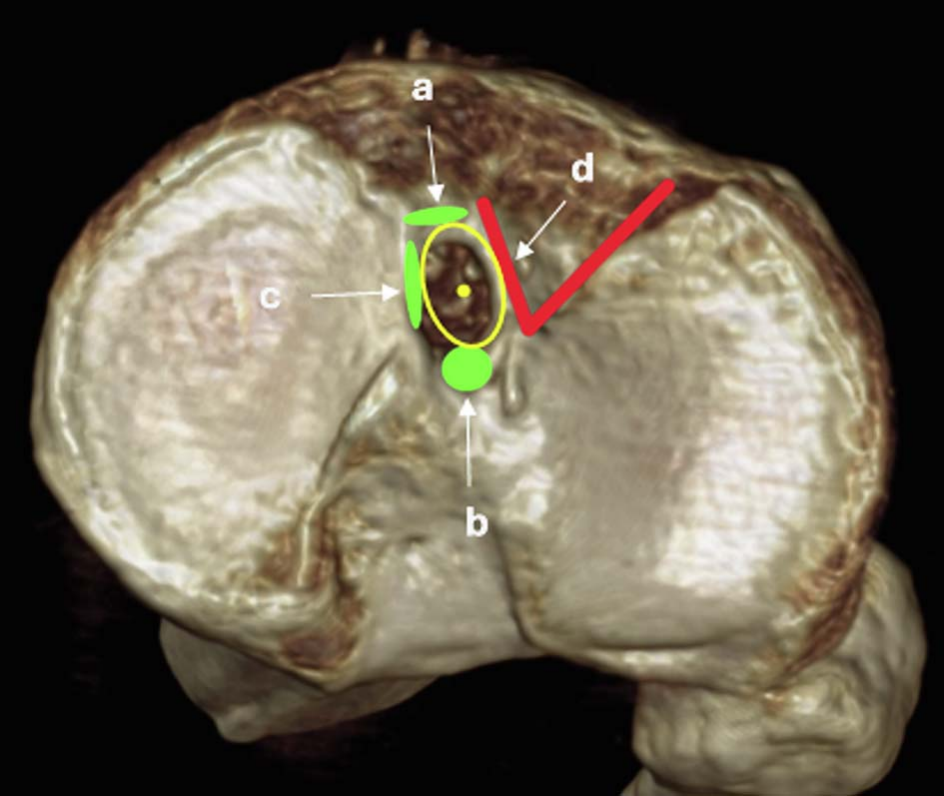
3-D CT scan reconstruction of a right tibial plateau axial view. Bony landmarks for ACL footprint and its center (yellow circle and yellow dot) are represented (a, anterior ridge; b, intertubercular fossae; c, medial intercondylar ridge; d, medial border of the lateral groove).

Intra-articular tibial tunnel position was analyzed using the intercondylar area method described by Cremer et al. [17] (Figure 1).

**Figure 2:**
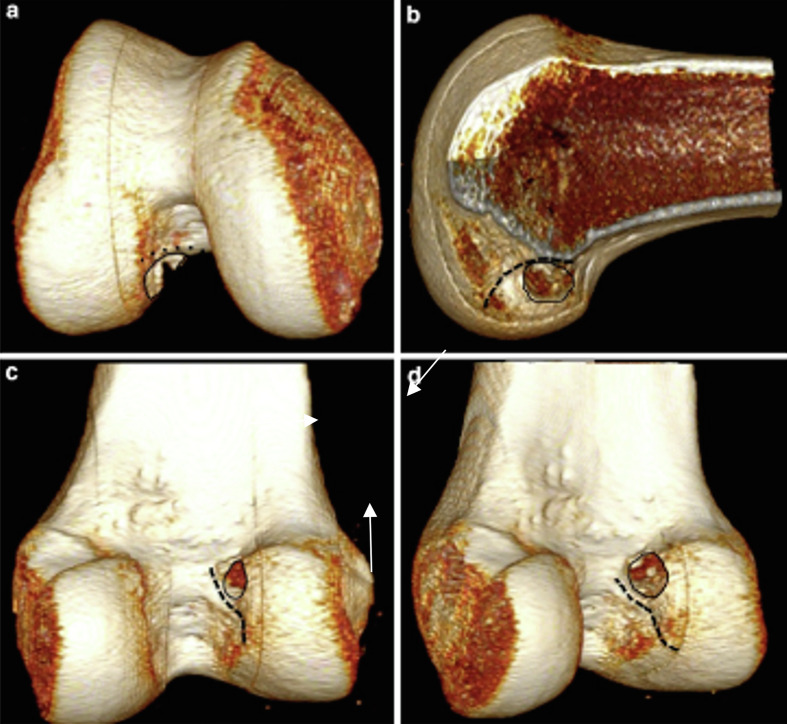
3-D rCT scan reconstructed images allow assessment of the intra-articular femoral tunnel aperture. a. Distal view. b. Medial view. c. Posterior view. d. Posteromedial view.

Tibial tunnel aperture was considered malpositioned if the anteroposterior (AP) gap between the center of the tunnel and the center of the ACL footprint was ≥ 5 mm or the mediolateral (ML) gap between the center of the tunnel and the center of the ACL footprint was ≥ 2 mm.

### Femoral tunnel position

**Figure 3:**
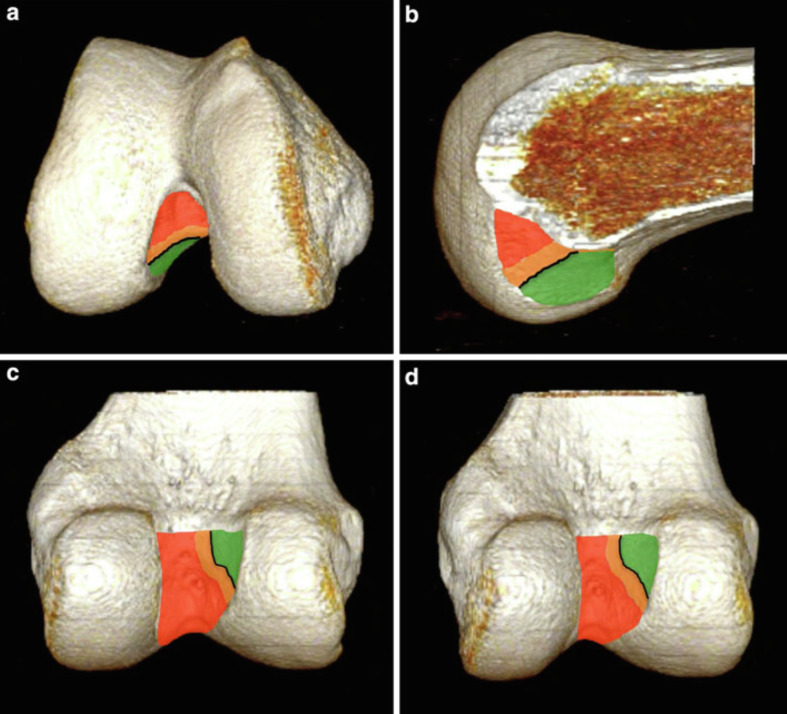
3-D CT scan reconstruction of the right distal femur illustrating the femoral tunnel classification system for the anterior cruciate ligament (ACL). a. Distal view. b. Medial view. c. Posterior view. d. Posteromedial view. *Type I* tunnels (appropriately positioned or anatomical) are entirely contained within the *green zone*, located posterior and inferior to the lateral intercondylar ridge (black line). *Type II* tunnels (slightly malpositioned) partially overlap the lateral intercondylar ridge and extend into the *orange zone*. *Type III* tunnels (severely malpositioned or non-anatomical) are positioned completely anterior and superior to the lateral intercondylar ridge, extending into the *red zone*.

Four 3-D reconstructed images were provided to allow assessment of the intra-articular femoral tunnel aperture (Figure 2). Intra-articular femoral tunnel position was analyzed using the technique described by Magnussen et al. [18]. The classification system is based on the location of the femoral tunnel relative to the lateral intercondylar ridge, with the knee flexed at 90° as during arthroscopy (Figure 3). The femoral tunnel was classified as type I, or anatomical, if it was located entirely below and posterior to the lateral intercondylar ridge as viewed from distally. The tunnel was classified as type II, or intermediate, if it was slightly malpositioned vertically, anteriorly, or both, such that the tunnel overlapped the lateral intercondylar ridge. The tunnel was classified as type III, or non-anatomical, if it was significantly malpositioned and located entirely anterior and/or vertical to the lateral intercondylar ridge. Tunnels type II and III (F) were considered malpositioned.

### Osteoarthritis and prognostic factors

Weight-bearing antero-posterior, schuss and lateral radiographs were obtained and assessed before the surgery and at the last follow-up for signs of OA according to the International Knee Documentation Committee (IKDC) classification [16]. Stages C and D indicating advanced stage of OA. Age at surgery, sex, injury-to-surgery time, body mass index (BMI), meniscectomy, type of sport, and cartilaginous status were also assessed.

### Ethical approval

All procedures were performed in accordance with the ethical standards of the institutional and/or national research committee, the 1964 Helsinki declaration and its later amendments, or comparable ethical standards. Data collection and analysis were carried out in accordance with MR004 Reference Methodology (Ref. 2238218v0) obtained on March 24, 2025. The study was registered and filed on the Health Data Hub website.

### Statistical analysis

Continuous variables were expressed as mean and standard deviation (SD) or as median and IQR, while categorical variables were presented as frequency distributions and percentages. The Shapiro–Wilk test was employed to evaluate the normality of the data. For comparisons between two groups, either the *t*-test or the Mann–Whitney U test was used, depending on whether the data followed a normal distribution. Categorical variables were analyzed using the chi-square test.

Finally, a multivariate analysis was carried out using a logistic regression model in order to calculate the Odds Ratio (OR) and its confidence intervals. A 95% confidence interval was applied, and statistical significance was defined as a *p*-value < 0.05.

All statistical analyses were performed using Python version 3.11 (Python Software Foundation, Wilmington, DE, USA) and the stats models library (v0.13).

## Results

### Femoral tunnel position

Of the 76 ACLRs performed, 53 (69,7%) were well-positioned, 17 (22.4%) were slightly malpositioned (overlapping the resident’s ridge), and 6 (7.9%) were significantly malpositioned (entirely vertical and/or anterior to the resident ridge). As such, 23 tunnels were considered malpositioned (30%) (type II and type III) (Table 2a).

**Table 2:** a. Distribution of femoral tunnel position. b. Distribution of tibial tunnel position

**a. Femoral tunnel position**		***N* = 76 (%)**
	Type I	53 (69.7%)
F	Type II	17 (22.4%)
	Type III	6 (7.9%)

**b. Tibial tunnel position**		***N* = 76 (%)**
Anatomic		39 (51.3%)
AP		13 (17.1%)
ML		15 (19.7%)
AP + ML		9 (11.8%)

AP: anteroposterior gap of the tibial tunnels ≥ 5 mm; ML: mediolateral gap of tibial tunnels ≥ 2 mm; F: femoral tunnels type II and III.

### Tibial tunnel position

Of the study group, 13 patients (17%) had an AP gap ≥ 5 mm and 15 (19%) a ML gap ≥ 2 mm. Association of AP and ML malposition was observed for 9 patients (11%). Global tunnels malposition (AP + ML + F) occurred for 4 patients (5%) (Table 2b).

### Osteoarthritis

The radiographs showed evidence of an advanced stage of OA in 18% of cases. At last follow-up, 60.5% of patients were IKDC A, 21.1% IKDC B, 10.5% IKDC C, and 7.9% IKDC D.

Global tunnel positions depending on the IKDC radiographic grade of OA are presented in Table 3.

**Table 3:** Tunnel positions depend on IKDC radiographic stage of OA.

	*N* (%)	IKDC radiographic stage of OA			
		A	B	C	D
**Analyzed Cohort**	76 (100%)	46 (60.5%)	16 (21.1%)	8 (10.6%)	6 (7.9%)
**Femoral Tunnel Position**					
Type I	53 (69.7%)	31	14	6	2
Type II	17 (22.4%)	13	1	1	2
Type III	6 (7.9%)	2	1	1	2
F (II + III)	23 (30.3%)	15	2	2	4
**Tibial Tunnel Position**					
Anatomic	39 (51.3%)	19	14	5	1
AP	13 (17.1%)	11	0	1	1
ML	15 (19.7%)	13	0	1	1
AP + ML	9 (11.8%)	3	2	1	3
**Combination of femoral and AP+ ML tibial tunnel malpositions (AP + ML + F)**	4 (5.3%)	0	1	0	3

AP: anteroposterior gap of the tibial tunnels ≥ 5 mm; ML: mediolateral gap of tibial tunnels ≥ 2 mm; F: femoral tunnels type II and III.

Significant association between AP+ML and AP+ML+F tunnels and the IKDC C/D group (*p* = 0.022 and *p* < 0.001, respectively) was found.

### Prognostic factors

The performance of a meniscectomy and the presence of cartilage lesions were significantly associated with an advanced radiographic stage of OA (*p* < 0.001 and *p* = 0.008, respectively). Distribution of independent risk factors is summarized in Table 4.

**Table 4:** Distribution of independent risk factors for osteoarthritis.

Factor	*N* (%)	*p*
Age at surgery > 30	40 (52.6%)	*0.709*
Male sex	40 (52.6%)	*0.338*
Injury-to-surgery time > 16 months	16 (22.2%)	*0.145*
BMI > 30	5 (6.6%)	*0.219*
Meniscectomy	18 (23.7%)	***<0.001***
Pivot sport	35 (46.1%)	*0.137*
Cartilage lesion	6 (7.9%)	***0.008***

Bold numbers mean that the result is significant.

## Discussion

The main finding of this study was that a non-anatomic position of the tibial tunnel and the combined malposition of both the tibial and femoral tunnels were associated with long-term radiographic OA. Isolated non-anatomic positioning of the femoral tunnel was not associated with OA in this study.

This study has some limitations. First, it has a retrospective design and a limited number of patients. Additionally, radiographs of the contralateral healthy limb were not available to compare the radiographic stage of OA, nor were long-leg films obtained to assess any pre-existing lower limb deformities. Cartilage lesions and the performance of concomitant meniscectomy were recorded as either present or absent, without grading severity or specifying compartmental location.

In this study, the 3D CT-scan tool was used to evaluate the positioning of the tunnels after ACLR. In 2005, Hoser et al. demonstrated that the 3D scanner was a reliable method in routine practice, compared to conventional radiographs, which showed low reliability (*p* = 0.22) [19].

Concerning tibial tunnels, 48% of the patients had non anatomic position in this study. This result can be explained by the complexity and variation of the footprint in itself. Pedneault et al., in their study on 40 patients, observed that the reconstructed tibial footprint was placed too anteriorly and medial to the ACL native footprint, while Shi et al. found that the average tunnel position was too lateral at the tibial side [14, 20]. Pedneault et al. found that the mean distance between the center of the native and reconstructed ACL at the tibial attachment site was 6.24 mm, and more than half the patients had less than 50% overlap with the native footprint [14]. The link between tibial tunnel malposition and OA can be explained by biomechanics. Anterior tibial tunnel placement significantly reduced anterior tibial translation and pivot shift movement, but may result in a roof impingement associated with a loss of extension and abrasion of the replacement graft, while excessively posterior tibial tunnel positioning fails to control laxity [15]. Contrary to anteroposterior graft placement, coronal tunnel location seems to have minimal effect on knee biomechanics [21]. The influence of sagittal positioning on the development of OA is discussed in the literature. Jin Hwan Ahn et al. highlighted in a 10-year mean follow-up study the importance of the sagittal tunnel position on the medial femoro-tibial compartment, while de Mees et al. found that tibial tunnel positions were not associated with radiographic OA [12, 13].

Concerning the femoral tunnel, approximatively 30% of them were considered as malpositioned in this study. In the literature, femoral tunnel placement appears to be a major key point for anatomic ACLR. Morgan et al. found that femoral tunnel malposition was cited in 219 (47.6%) of 460 revision cases [22]. Seo et al. found that 30% of femoral tunnel placement was too anterior, resulting in excessive residual laxity [23]. Shi et al. found that, at the femoral side, the average tunnel position in the ACLR failure group was significantly more anterior and superior [20]. Moreover, the specific risk of meniscal surgery after ACLR appears to be increased after femoral tunnel malposition [24]. In this study, isolated femoral tunnel malpositioning was not associated with an advanced stage of OA. This result is consistent with studies with a long-term follow-up [14]. It can be explained by the low number of type III tunnels (7.9%) and the technique used to perform femoral tunnels. Osti et al. showed that anteromedial portal and outside-in surgical techniques were superior in positioning the ACL femoral tunnel at the center of the native ACL attachment site compared with the transtibial technique [15].

In this study, the advanced stage of OA was found in 18% of cases, which is similar to studies with the same follow-up [14]. Associated injuries, especially medial and lateral meniscectomy and cartilage lesions, play a major role in OA in this study, which has also been widely found in the literature [25].

## Conclusion

The malpositioning of tibial tunnels, specifically the combination of ML and AP displacement, is significantly associated with an advanced radiographic stage of OA at a follow-up of ten years, regardless of femoral tunnel malposition.

Additionally, undergoing a meniscectomy or identifying a cartilage lesion during ACLR has been identified as a risk factor for an advanced radiographic stage of OA at a follow-up of ten years.

## Data Availability

The data that support the findings of this study are not openly available due to reasons of sensitivity and are available from the corresponding author upon reasonable request.
